# Trends in colorectal cancer incidence among younger adults—Disparities by age, sex, race, ethnicity, and subsite

**DOI:** 10.1002/cam4.1621

**Published:** 2018-06-22

**Authors:** Amanda B. Crosbie, Lisa M. Roche, Linda M. Johnson, Karen S. Pawlish, Lisa E. Paddock, Antoinette M. Stroup

**Affiliations:** ^1^ Cancer Epidemiology Services New Jersey Department of Health Trenton NJ USA; ^2^ Rutgers Cancer Institute of New Jersey New Brunswick NJ USA; ^3^ Department of Epidemiology Rutgers School of Public Health Piscataway NJ USA

**Keywords:** colorectal cancer incidence, colorectal cancer subsites, disparities, time trends, younger adults

## Abstract

Millennials (ages 18‐35) are now the largest living generation in the US, making it important to understand and characterize the rising trend of colorectal cancer incidence in this population, as well as other younger generations of Americans. Data from the New Jersey State Cancer Registry (n = 181 909) and Surveillance, Epidemiology, and End Results program (n = 448 714) were used to analyze invasive CRC incidence trends from 1979 to 2014. Age, sex, race, ethnicity, subsite, and stage differences between younger adults (20‐49) and screening age adults (≥50) in New Jersey (NJ) were examined using chi‐square; and, we compared secular trends in NJ to the United States (US). Whites, men, and the youngest adults (ages 20‐39) are experiencing greater APCs in rectal cancer incidence. Rates among younger black adults, overall, were consistently higher in both NJ and the US over time. When compared to older adults, younger adults with CRC in NJ were more likely to be: diagnosed at the late stage, diagnosed with rectal cancer, male, non‐white, and Hispanic. Invasive CRC incidence trends among younger adults were found to vary by age, sex, race, ethnicity, and subsite. Large, case‐level, studies are needed to understand the role of genetics, human papillomavirus (HPV), and cultural and behavioral factors in the rise of CRC among younger adults. Provider and public education about CRC risk factors will also be important for preventing and reversing the increasing CRC trend in younger adults.

## 1 INTRODUCTION

Recent studies indicate colorectal cancer (CRC) has been rising in younger adults who are under the recommended screening age of 50.^1^, ^2^, ^3^, ^4^, ^5^, ^6^, ^7^ In New Jersey (NJ), an average of 435 younger adults (approximately 10% of all CRC cases) are diagnosed each year.^8^ The latest research, using age‐period‐cohort modeling, found that US adults born around 1990 have double the risk of colon cancers and quadruple the risk of rectal cancer compared to adults born around 1950.^1^ Prior studies have analyzed race/ethnicity and sex,^1^, ^2^, ^3^, ^4^, ^5^, ^6^ but the authors are not aware of studies in NJ or the US which have examined trends by age, sex, race, ethnicity, and subsite in combination in younger adults with CRC. Right‐ and left‐sided colon cancers have markedly different underlying biological characteristics, and are distinct from rectal cancers. This has important implications for disease severity, choice of therapies and prognosis,^9^, ^10^, ^11^ which makes it very important to understand who is most at risk for CRC in younger adulthood in today's population.

NJ's unique population demographics and high population density^12^ make the registry data a valuable tool for comparisons to the US to further define those most at risk for CRC in younger adulthood. We have considerably fewer Hispanics who report being of Mexican descent (NJ: 14% vs US: 63%),^12^ and we are the most densely populated state in the US (NJ: 1195.5 per sq. mile vs US: 87.4 per sq. mile in the US), which has ramifications to the burden of disease, as well as ensures our numbers for comparisons. These differences provide insight into the potential influences of cultural practices on current trends, with the purpose of encouraging other registries to consider how their population is different, and to clarify what demographic and clinical features are behind the trend changes.

Many factors are known to be associated with CRC including genetic syndromes, a personal or family history of CRC or adenomatous polyps, a personal history of chronic inflammatory bowel disease such as ulcerative colitis or Crohn’s disease, and diabetes.^13^ Other modifiable risk factors include physical inactivity, obesity or being overweight, high consumption of red and/or processed meats, smoking, and moderate‐to‐heavy alcohol consumption (2‐4 drinks a day).^13^ Various studies indicate that some of these risk factors affect CRC subsites differently (Table S1).

The health attributes of younger adults, and how these behaviors have changed overtime are also important to consider. For example, a higher proportion of younger adults (ages 20‐49) report current smoking behaviors compared to screening age adults (18% vs 12%, respectively) (BFRSS). Episodic heavy alcohol use or “binge drinking” became the number one health problem affecting college students in the 1990s; a behavior which does not appear to have changed over time.^14^, ^15^ Binge drinking remains higher in younger adults at 23% compared to 9% for screening age adults; the highest proportion of self‐reported binge drinking, occurring in those ages between 20 and 29 (30%).^16^ Recent birth cohorts in the US show that younger generations are reaching a higher prevalence of obesity earlier in life, resulting in a greater duration and degree of obesity in their lifetime.^17^ In NJ, 25% of younger adults self‐report being obese, compared to 30% of screening age adults.^16^ NJ ranks 36th out of 50 in obesity, making obesity prevalence considerably lower than other states.^18^

The role of sexually transmitted infections (STIs) and risk‐taking behaviors in CRC development remains unclear. A recent review reported a higher prevalence of HPV in CRC tumors than noncancerous tissue.^19^ An earlier review of HPV and CRC also concluded that HPV may be associated with a subset of CRC.^20^ In terms of transmission, Mosher et al,^21^ found that 40% of men and 35% of women (ages 25‐44) self‐report having had anal sex with an opposite sex partner, and about 6.5% of men have had oral or anal sex with another man. The San Francisco Men's Health Study also found that younger men were more likely to engage in unprotected anal intercourse compared to older men.^22^

Millennials (ages 18‐35) are now the US's largest living generation at 79.8 million,^23^ making it important to understand and characterize the subgroups of CRC in this population, and other younger generations of Americans. The purpose of this study was to examine secular trends in invasive CRC incidence overall, as well as by subsite (proximal colon, distal colon, and rectal) and key demographic factors among NJ younger adults ages 20‐49 years. We also compared CRC incidence among younger adults in NJ to the US, and NJ younger adults to older adults (≥50 years) diagnosed with CRC.

## 2 METHODS

The New Jersey State Cancer Registry (NJSCR), is a population‐based cancer incidence registry established in October 1978 to monitor cancer among the more than 8.9 million residents of NJ.^12^ NJSCR is a Surveillance, Epidemiology, and End Results (SEER) expansion registry and a participant in the Centers for Disease Control and Prevention's (CDC) National Program of Cancer Registries (NPCR). The NJSCR has received recognition consistently from NCI, CDC, and the North American Association of Central Cancer Registries (NAACCR) for its high quality and timely submission of data. Demographic and clinical information about each newly diagnosed cancer case (eg cancer site, age, sex, race, ethnicity, date of diagnosis) for this study were extracted from the NJSCR.

Between 1979 and 2014, a total of 181 909 invasive CRC cases in NJ ages 20 and older were included in the analyses, after excluding 5364 (2.9%) cases ascertained only from death certificate and autopsy reports. Of these cases, 12 080 (6.6%) were 20‐49 and 169 829 (93.4%) were 50 and older. Between 1992 and 2014, 113 501 invasive CRC cases were included in the analyses. Of these cases, 8588 (7.6%) were 20‐49 and 104 913 (92.4%) were 50 and older. The stage analyses included 123 420 cases (both in situ and invasive CRC); 9217 (7.5%) were 20‐49, and 114 203 (92.5%) were 50 and older.

CRC sites were based on primary site and histology coded to the International Classification of Diseases for Oncology, 3rd Edition (ICD‐O‐3).^24^ CRC sites included C18.0, 18.2‐20.9, and C26.0; diagnosis of appendix (C18.1) was excluded. ICD‐O‐3 Histological types 9590‐9989, 9050‐9055, and 9140 were also excluded. Cases with unknown age or county of residence were excluded, as were cases reported only through autopsy reports or death certificates. CRC subsites were defined as: *proximal* includes the cecum, ascending colon, hepatic flexure, and transverse colon (C18.0, C18.2‐18.4), *distal* includes the splenic flexure, descending and sigmoid colon (C18.5‐C18.7), and *rectal* which includes the rectosigmoid junction and rectum (C19.9, C20.9). Large intestine, NOS (C18.8‐18.9, 26.0), was excluded from the subsite‐specific analyses.

SEER*Stat software (version 8.3.4, NCI)^25^ was used to generate frequency counts, percentages, and annual age‐adjusted incidence rates with 95% confidence intervals (CIs) by age, sex, race, ethnicity, CRC subsite, and stage. Analogous data from nine SEER population‐based cancer registries (Atlanta, Connecticut, Detroit, Hawaii, Iowa, New Mexico, Seattle‐Puget Sound, San Francisco‐ Oakland, Utah)^26^ were used to compare NJ to the US between 1979 and 2014 by sex and age (n = 448 714). Of these, 34 333 (7.7%) were 20‐49 and 414 381 (92.3%) were 50 and older. For race and ethnicity, analogous data from 13 SEER registries (SEER 9 plus San Jose‐Monterey, Los Angeles, Alaska Native Registry, rural Georgia)^27^ were used to compare NJ to the US between 1992 and 2014 (n = 401 739). In this database, 35 898 (8.9%) were 20‐49 and 365 841 (91.1%) were 50 and older. SEER 13 registries reported on expanded race/ethnicity starting in 1992, which allowed us to include Hispanic and Asian or Pacific Islanders (APIs) in our race/ethnicity subanalyses. Age‐adjusted rates were based on the 2000 US population standard.

The most recent cohort of CRC cases (1992‐2014) were imported into SAS version 9.4^28^ to identify any significant differences by age group (20‐49 vs ≥50) using Pearson's chi‐square. Comparisons were made by sex, race, ethnicity, subsite, and stage. Stage included in situ cases, which added 9919 cases to this sub category; 6.3% ages 20‐49 (n = 629), and 93.7% (n = 9290) ages 50 and older. Time trends stratified by 10‐year age groups (20‐29, 30‐39, 40‐49, 50‐59 etc.), age below and at/above screening age (20‐49, ≥50), sex, race, ethnicity, and subsite were analyzed using JoinPoint Regression Program (version 4.4.0.0, NCI),^29^, ^30^ which calculates annual percent changes (APCs) and identifies points in time when the APCs change in direction and/or velocity.

Time trends by CRC subsite were also explored for NJ from 1979 to 2014 in younger adults by three age groups (20‐39, 40‐49, and 20‐49), sex, and race (white, black). Younger adults in the 20‐29 age group year were combined with the 30‐39 age group for the subsite analyses because of small numbers in the 20‐29 age group. API and Hispanics were excluded from these analyses due to small numbers in the subcategories by subsite, and the lack of population data for these racial/ethnic subgroups prior to 1990 in NJ. Statistical significance for all analyses was set at *P* < .05.

## 3 RESULTS

Between 1992 and 2014, a significantly larger proportion of younger CRC patients were male (53.3%) compared to CRC patients ages 50 and older (49.7%) (Table 1). Younger adults with CRC were also more likely to be black (16.8% vs 10.4%), API (5.6% vs 2.2%) and Hispanic of any race (13.1% vs 6.0%) compared to those of screening age (≥50). Subsite differences were pronounced between age groups, with higher proportions of rectal (39.5% vs 27.7%) and distal colon (43.9% vs 33.8%) cancers in the younger adults, and higher proportions of proximal colon cancers in older adults (58.2% vs 48.2%). Unfortunately, almost two‐thirds of younger adults were diagnosed at the late stage (57.7%), which is significantly higher than screening age adults (50.7%).

**Table 1:** Colorectal cancer case characteristics by age group and time period in NJ, 1992‐2014

Characteristic	Age group, y		*P*‐value
	Ages 20‐49	Ages ≥50	
Sex	N = 8588 (100%)	N = 104 913 (100%)	
Female	4010 (46.7%)	52 721 (50.3%)	
Male	4578 (53.3%)	52 192 (49.7%)	<.0001
Race			
White	6542 (76.2%)	91 251 (87.0%)	
Black	1444 (16.8%)	10 912 (10.4%)	
API	477 (5.6%)	2263 (2.2%)	
Other	125 (1.5%)	487 (0.5%)	<.0001
Ethnicity			
Hispanic (of any race)	1125 (13.1%)	6279 (6.0%)	
Not Hispanic (of any race)	7463 (86.9%)	98 634 (94.0%)	<.0001
Cancer subsite			
Colon	5192 (60.5%)	75 882 (72.3%)	
Proximal	2286 (44.0%)	41 606 (54.8%)	
Distal	2499 (48.1%)	28 161 (37.1%)	
Other	407 (7.8%)	6115 (8.1%)	*<.0001*
Rectal	3396 (39.5%)	29 031 (27.7%)	<.0001
Stage^a^			
1992‐2014	n = 9217	n = 114 203	
Early^b^	3278 (35.6%)	46 639 (40.8%)	
Late^c^	5320 (57.7%)	57 905 (50.7%)	
Unstaged	619 (6.7%)	9659 (8.5%)	<.0001

Percentages do not always add up to 100% due to rounding.

a Includes in situ.

b Early stage is defined as in situ and local stages.

c Late stage includes regional and distant stages.

Time trend analyses revealed that NJ incidence rates of invasive CRC among younger adults were consistently higher than US rates up to about 2009 when rates began to converge (Figure S1A, Table S2). By the mid‐1990s, younger adults (20‐49) in both NJ and the US began experiencing significant increases in CRC incidence (NJ APC: +1.1, 95% CI: +0.6‐1.6; US APC: +1.8, 95% CI: +1.5‐2.1). The rate increases were most notable among the youngest age group of 20‐29 years (NJ APC: +2.1, 95% CI: +1.3‐3.0; US APC: +2.6, 95% CI: +2.0‐3.1) (Figure S1C, Table S2). Rates are, however, still lowest in those ages 20‐29, with an age‐adjusted average annual incidence rate between 1979 and 2014 of 1.3 per 100 000 (95% CI: 1.2‐1.4) in NJ, and 1.1 per 100 000 (95% CI: 1.1‐1.2) in the US. Interestingly, US younger adults ages 30‐39 and 40‐49 experienced declines in CRC incidence until the early 1990s, at which time rate changes occurred causing an up spike in CRC incidence at a faster pace than NJ for these younger adults. The US APC for adults ages 30‐39 from 1988 to 2014 was +2.0 (95% CI: 1.6‐2.4), and for US younger adults ages 40‐49, between 1994 and 1996, the APC was +1.6 (95% CI: 1.3‐2.0). NJ 30‐39 years olds had a steady increase of +1.1 (95% CI: 0.6‐1.6) from 1979 to 2014, and 40‐49 years olds did not demonstrate any significant rate changes +0.1 (95% CI: −0.2 to +0.3) from 1979 to 2014 (Figure S1C,D, Table S2).

By contrast, older adults have been experiencing significant declines in CRC from the mid to late 1980s, which is attributable to improvements in screening; colonoscopy with the removal of colorectal polyps began in 1969.^31^ The most notable declines were seen in those of oldest ages (80+), whose incidence is highest (Figure S1I, Table S2). Declines have been less pronounced in those ages 50‐59 which is concerning, and supports the idea that the elevated risk in younger adults will carry forward into older age. Since 1988, the APC in US adults ages 50‐59 has been a slow and steady −0.5% (95% CI: −0.7 to −0.3) decline. For NJ adults ages 50‐59, a non‐significant increase of +2.0% (95% CI: −0.5 to +4.5) began in 2010.

From 1979 to 2014, CRC rates increased steadily in younger NJ men ages 20‐49 (APC: +0.5, 95% CI: +0.3‐0.8), whereas rates in their US counterparts, only began to increase around 1993 (APC: +1.7, 95% CI: +1.4‐2.0) (Figure S2A, Table S3). Trends among younger NJ and US women were similar in that increasing rates began around the mid‐1990s; however, US rates are increasing at a higher velocity than NJ (NJ APC: +1.2, 95% CI: +0.3‐2.0 vs US APC: +1.8, 95% CI: +1.3‐2.3) (Figure S2B, Table S3). Rates remain higher in men compared to women for both NJ and the US. The average annual incidence rate for NJ men between 1979 and 2014 was 10.7 per 100 000 (95% CI: 10.4‐11.0). For NJ women, the average annual incidence rate after the increases began (between 1994 and 2014) was 8.9 per 100 000 (95% CI: 8.7‐9.01).

**Figure 1:**
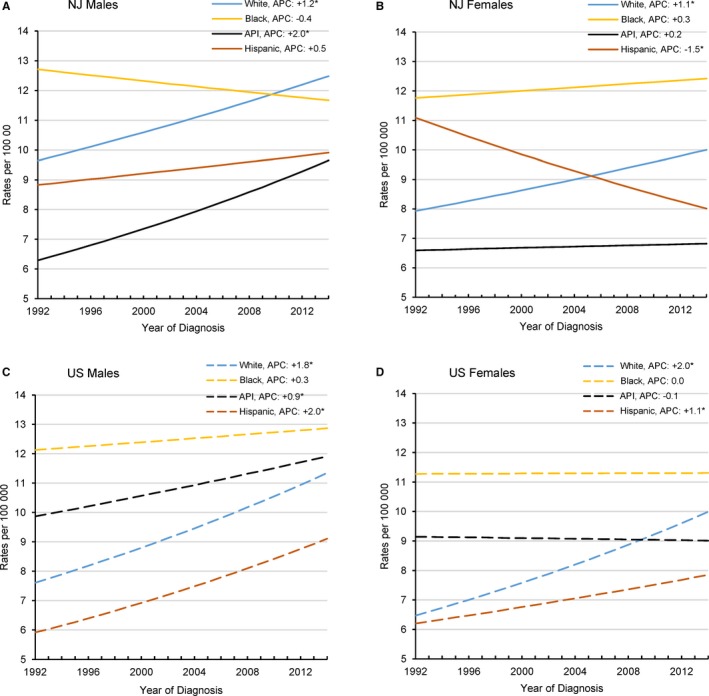
Annual Percent Change (APC) in younger adults (20‐49 years); colorectal cancer incidence rates by race/ethnicity and year, 1992‐2014. (A) NJ Males, (B) NJ Females, (C) US Males, and (D) US Females. API, Asian or Pacific Islander. Persons of Hispanic ethnicity may be of any race or combination of races. The categories of race and ethnicity are not mutually exclusive. Rates are per 100 000 and age adjusted to the 2000 US Standard Population (19 age groups ‐ Census P25‐1130). An asterisk denotes that the APC is significant (*P* < .05)

Of primary interest were any significant differences by race/ethnicity and sex in younger adults. Surprisingly, we found little consistency between NJ and the US in the race/ethnicity subanalyses (Figure 1, Table S4). We did observe that younger black men and women had consistently higher rates with little to no change over time in both NJ and the US, while rates in whites significantly increased. The rate velocity in younger NJ API men was twice that of their US counterparts (APC: +2.0, 95% CI: +0.0‐3.9 and +0.9, 95% CI: +0.2‐1.5, respectively). Although, caution must be exercised in this interpretation because of small numbers in the NJ API subgroup (n = 254). Unlike CRC rates among younger US Hispanic women, which have been increasing since 1992 (APC: +1.1, 95% CI: +0.5‐1.6), corresponding NJ rates have been declining significantly (APC: −1.5, 95% CI: −2.9 to 0.0). Rates overall between 1992 and 2014 were higher in NJ Hispanic women, however, at 9.0 per 100 000 (95% CI: 8.3‐9.8), compared to US Hispanic women (7.1 per 10 000, 95% CI: 6.8‐7.4). Average annual incidence rates between 1992 and 2014 were highest in black men and similar between NJ and the US (12.0 per 100 000 (95% CI: 11.1‐12.9; 12.5 per 100 000, 95% CI: 12.0‐13.0, respectively). Black women had the next highest average annual incidence rates for the time period (NJ: 11.9 per 100 000, 95% CI: 11.1‐12.8; US: 11.2 per 100 000, 95% CI: 10.8‐11.7), followed by US API men (10.9 per 100 000, 95% CI: 10.5‐11.4) and NJ white men (10.9 per 100 000, 95% CI).

**Figure 2:**
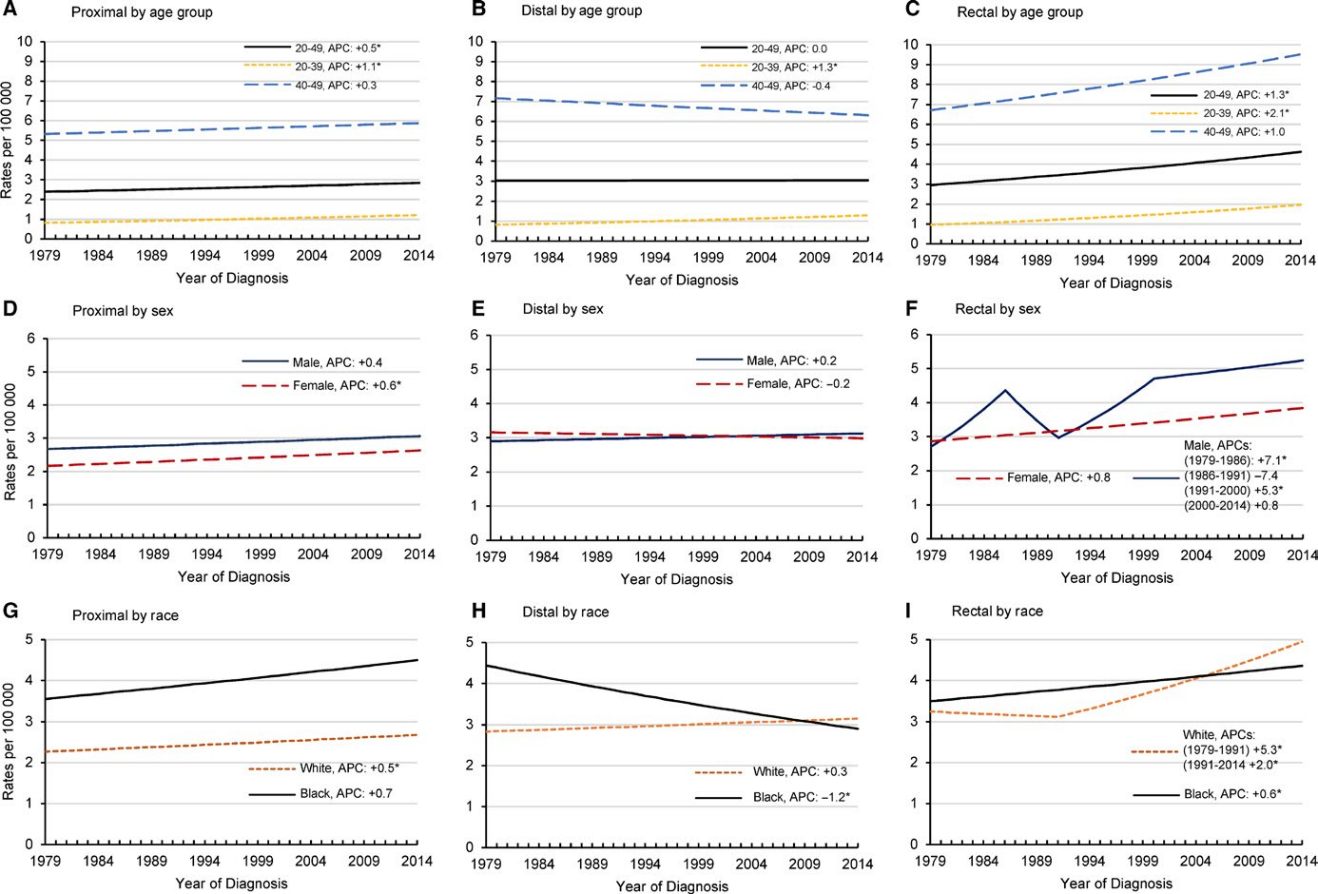
Annual Percent Change (APC) in younger adults (20‐49 years); invasive colorectal cancer incidence rates by subsite, age group, sex, race, and year in NJ, 1979‐2014. The scale of the *y*‐axis varies to depict the trends. Rates are age adjusted to the 2000 US Standard Population (19 age groups ‐ Census P25‐1130). An asterisk denotes that the APC is significant (*P* < .05)

Between 1979 and 2014, the average annual incidence rates for all CRC subsites remained highest in the oldest NJ younger adults (40‐49), and highest for the rectal subsite in all younger age categories (Figure 2A‐C, Table S5). For those ages 40‐49, the incidence rates were 5.6 per 100 000 (95% CI: 5.3‐5.8), 6.6 per 100 000 (95% CI: 6.4‐6.9), and 8.1 per 100 000 (95% CI: 7.9‐8.4), for proximal, distal and rectal subsites, respectively. The rate for rectal cancer in 20‐39 year olds was 1.4 per 100 000 (95% CI: 1.3‐1.5), compared to 1.0 per 100 000 (95% CI: 0.9‐1.1) for both the proximal and distal subsites.

CRC is still rare in younger adults, but the changing velocities in the rates over time, especially for rectal cancer, are concerning. Rectal cancer increased at a significantly faster pace between 1979 and 2014 (20‐49, APC: +1.3, 95% CI: 0.9‐1.6 per year), compared to proximal and distal colon cancers (20‐49,APC: +0.4, 95% CI: +0.1‐0.8, and 0.0, ns, respectively); and, the trend was more pronounced in 20‐39 years olds (APC: +2.1, 95% CI: +1.4‐2.7).

Gender‐specific subsite analyses in younger adults showed greater differences in the rectal subsite compared to proximal and distal subsites (Figure 2D‐F, Table S5). NJ women have experienced steady increases in rectal cancers (APC from 1979 to 2014: +0.8, 95% CI: +0.3‐1.4), whereas NJ men after several periods of more rapid increases over time (APC from 1979 to 1986: +7.1, 95% CI: +2.0‐12.4; APC from 1991 to 2000: +5.3, 95% CI: +2.0‐8.7), have been steadily increasing since 2000 (APC: +0.8, ns). At the start of the trend changes for NJ men (1979‐1986), the average annual age‐adjusted incidence rate was 3.5 per 100 000 (95% CI: 3.1‐3.9). In the most recent time trend of 2000‐2014, the average annual incidence rate for NJ men (20‐49) was 5.0 per 100 000 (95% CI: 4.7‐5.3). The incidence rate for NJ younger women between 1979 and 2014 was 3.3 per 100 000 (95% CI: 3.2‐3.5).

Black/white comparisons showed steady increases in rectal cancer among younger NJ blacks (APC: +0.6, 95% CI: +0.0‐1.3), but starting around 1991 rates among NJ whites increased at a faster pace (APC: +2.0, 95% CI: 1.3‐2.7), and eventually surpassed NJ blacks around 2005 (Figure 2I, Table S5). When examining the average annual age‐adjusted incidence rates, between 1979 and 2014 rectal cancer in blacks was 3.9 per 100 000 (95% CI: 3.6‐4.2). The latest trend for whites starting in 1991 has an average annual age‐adjusted incidence rate equal to the rate seen in blacks (3.9 per 100 000, 95% CI: 3.8‐4.1).

Between 1979 and 2014, in addition to 40‐49 year olds, proximal cancers were also highest in blacks (1979‐2014, 4.0 per 100 000, 95% CI: 3.7‐4.3), and men (2.9 per 100 000 95% CI: 2.7‐3.0) (Figure 2A,D,G, Table S5). Proximal cancers showed small and steady increases overtime for all age group, although it was not significant in 40‐49 year olds. Ages 20‐39 experienced a significant increase in proximal colon cancers (APC: +1.1 (9%% CI: +0.5‐1.8), as have NJ younger women (APC: +0.6, 95% CI: +0.1‐1.1). NJ younger men have had a non‐significant rise in proximal colon cancers (APC: +0.4, ns).

Curiously, distal cancers have significantly declined in blacks since 1979 (APC: −1.2, 95% CI: −2.1 to −0.3) (Figure 2H, Table S5). This is offset against a significant increase in 20‐39 year olds (APC: +1.3, 95% CI: +0.6‐2.1). Examination of the distal subsite by sex (Figure 2E, Table S5) revealed little change overtime, although rates for younger men and women appear to have “flip flopped” in recent years, with parallel age‐adjusted average annual incidence rates for the time frame of 1979‐2014 between the two sexes (3.0 per 100 000, 95% CI: 2.9‐3.1 for women; 3.0 per 100 000, 95% CI: 2.8‐3.1 for men).

Ultimately, what this means for New Jersey is about 100 more cases of CRC in younger adults in 2014 compared to 1992, with rate changes occurring at faster paces in 20‐39 year olds, men, and rectal cancer.

## 4 DISCUSSION

We found that age‐adjusted invasive CRC incidence rates in younger adults (20‐49) have risen significantly since the mid‐1990s in NJ, similar to the increase in US younger adults.^1^, ^2^, ^3^, ^4^, ^5^, ^6^ This contrasts with older adults (≥50) in NJ and the US whose CRC incidence rates have declined significantly for several decades.^1^, ^2^, ^3^, ^4^, ^5^, ^6^ CRC incidence rates are increasing the fastest among the youngest adults ages 20‐39. Since rates have risen similarly for CRC diagnosed at the early and late stages, it is unlikely that the increase in CRC among younger adults is due to improvements in diagnosis.^1^, ^5^ Furthermore, a recent letter to JAMA by Siegel et al,^32^ identified increases in mortality in younger adults with CRC, confined to whites, which they used as an indication of a true rise in CRC incidence, especially since we know whites are driving the incidence changes. These findings are cause for concern because over time, as younger adults age, their risk for subsequent CRC increases,^33^ and their need for ongoing surveillance (ie screening) will be a growing public health issue due to the size of the Millennial population. Indeed, if CRC incidence in this cohort increases, the declines that we see in NJ due to effective screening and prevention efforts may slow and perhaps reverse direction.

Obesity (and the behaviors that drive weight gain) is considered a likely culprit for the increase in CRC among younger adults because the rise in obesity prevalence parallels the rise in CRC in the US.^1^, ^34^, ^35^ However, obesity has been associated most strongly with distal colon cancers,^36^ which is the subsite that remained stable among younger NJ adults. Thus, increasing obesity does not completely account for the sharp increase in rectal cancer among younger adults.^6^

Elevated CRC rates among younger adult blacks in both NJ and the US, and the relative stability of the rates over time suggest that modifiable behavioral risk factors related to CRC (ie physical inactivity, obesity, diabetes)^37^, ^38^, ^39^, ^40^ with known higher prevalence in the black community may not completely explain the recent increases in CRC in younger adults.^1^, ^41^, ^42^ Unfortunately, our subsite analyses were limited to whites and blacks, and, therefore, were not able to assess trends by subsite in API or Hispanics. Including these populations in future analyses may shed further light on the impact of socioeconomic, cultural, and behavioral influences on CRC incidence.

Although research is scarce in this area, there is a growing body of literature which outlines the increasing prevalence of anal sexual practices among younger adults, with the peak in 30‐34 year olds.^21^, ^22^, ^43^ Studies indicate engagement in anal sexual behaviors has increased over time, with some lifetime prevalence estimates for heterosexual anal intercourse as high as 40%.^21^, ^22^, ^43^ There is also suggestion that condom use with anal sex compared to vaginal sex is lower in heterosexuals. The physical implications of anal sex include trauma to the anus and rectum, as well as inflammatory responses to cleansers, lubricants, semen and/or STIs.^43^ There are also studies which physiologically link HPV to CRC tumors.^31^, ^32^

Our analyses indicate that CRC is still rare in younger adults, but the trend changes are concerning. It is thus important to consider the behavioral attributes which may be changing in younger adult culture. We know they drink more,^14^, ^15^, ^16^ and they engage in riskier sexual behaviors^21^, ^22^ than earlier generations. HPV is associated with cancers in other organs,^44^ and we know that alcohol has a greater impact on the rectal subsite.^45^ The proximity of the rectum to the anus, and the known oncogenic association of HPV with anal cancer^46^ provides a possible role for STIs in the rising rectal cancer trends. Given IBD and other causes of bowel irritation are risk factors for CRC,^13^ it seems feasible to propose that HPV or another STI may be playing a role in the trend changes.

Unfortunately, the registry does not collect HPV status on cancer cases. HPV is also extremely common, and is not reportable to health departments,^47^ making it hard to understand the true burden of disease. However, there is general agreement across multiple studies that genital HPV prevalence decreases with age, independent of sexual behavior and multiple partners, especially in women,^48^, ^49^, ^50^ making it worth exploring as a possible causative agent when you consider the CRC trends changes are most apparent in the youngest younger adults, whites, and men. HPV could be changing in this subset of the population due to increased sexual risk taking, and other dangerous health behaviors such as excessive drinking. Understanding the timeline of evolving sexual practices, and the characteristics of those who engage in anal sexual behaviors and subsequent increased risks from inflammation and/or infection and its implications to rectal health will need to be evaluated to understand whether this is a realistic and pertinent factor in the rise in rectal cancers in younger adults.

The differences in CRC incidence rates between NJ and US Hispanics may be a function of what we broadly define as “Hispanic”. In 2015, 27.9% of the estimated Hispanic population in NJ self‐identified as Puerto Rican, compared to 9.2% for the US. Most Hispanics in the US self‐identified as Mexican, 63.0%, vs only 14.0% in NJ.^12^ These differences in country of origin may account for some of the differences in the prevalence of CRC risk factors, including obesity and diabetes between NJ vs US Hispanics. Different origins between NJ and US blacks and APIs may also explain the racial differences in CRC rates. Of note, 41% of NJ's Asian population self‐identify as Asian Indian compared to the US at 20%, and a higher proportion of NJ blacks are foreign born, 17%, compared to the US, 9%.^12^

### 4.1 Limitations and strengths

One study limitation, common to time trend analyses using cancer registry data, is that the most recent few years of data may be incomplete due to reporting delays. This may result in underestimates of incidence rates and underestimates of APCs, which appear to level off from 2012 to 2014. We may have also lacked the power to detect significant changes in rates over time in some of our subgroup analyses due to small numbers. There is potential for anal cancers to be misclassified as rectal SSC. However, rectal SSC comprises <0.5% of the NJ CRC data in younger adults, making the effects of any misclassification minimal.

## 5 CONCLUSIONS

Given the large burden on our society from CRC, additional research is needed to identify the root causes and etiology of younger onset CRC; and, to discern if, and to what extent, genetic, cultural, and behavioral factors play a role in the increasing CRC risk in younger adults. Surveillance studies such as ours provide clues, but are limited in ability to identify the causative factors; further epidemiologic studies (case control, cohort) are needed.

Careful studies of the risks and benefits of lowering the current screening age, perhaps specifically for low‐cost, low‐risk methods such as fecal immunochemical test kits or fecal DNA tests, should also be considered.^2^ Surveillance among survivors will then be key to understanding prevention and/or early detection of subsequent primaries. In the meantime, public and clinician awareness could promote early stage diagnoses in symptomatic younger adults.

## CONFLICT OF INTEREST

None declared.
