# Supplementary figures and images for: Trends in colorectal cancer incidence among younger adults—Disparities by age, sex, race, ethnicity, and subsite
Source: Cancer Med. 2018 Jun 22;7(8):4077–86. doi: 10.1002/cam4.1621 (PMC6089150; doi:10.1002/cam4.1621)

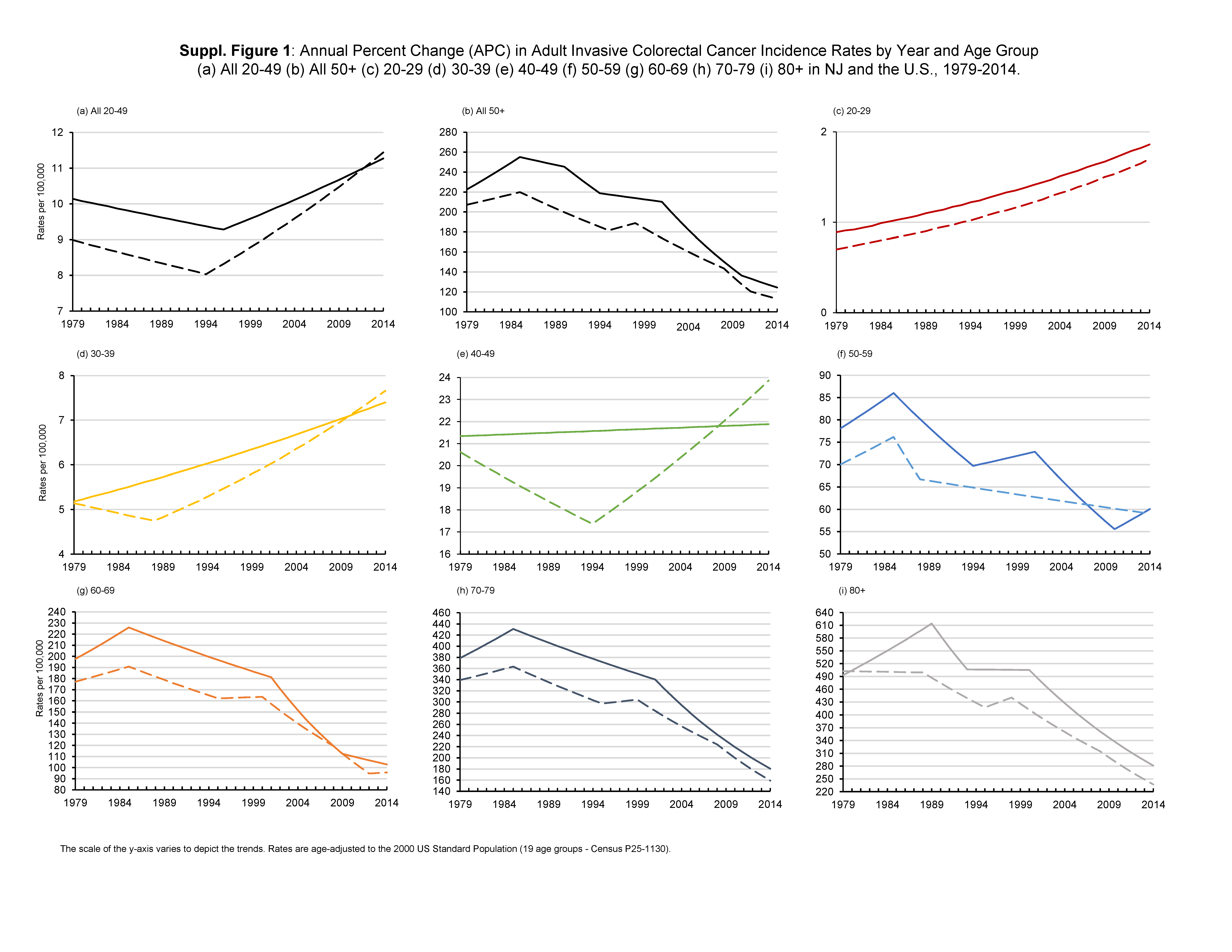

Supplement: Supplementary file 1 [file CAM4-7-4077-s001.tif]

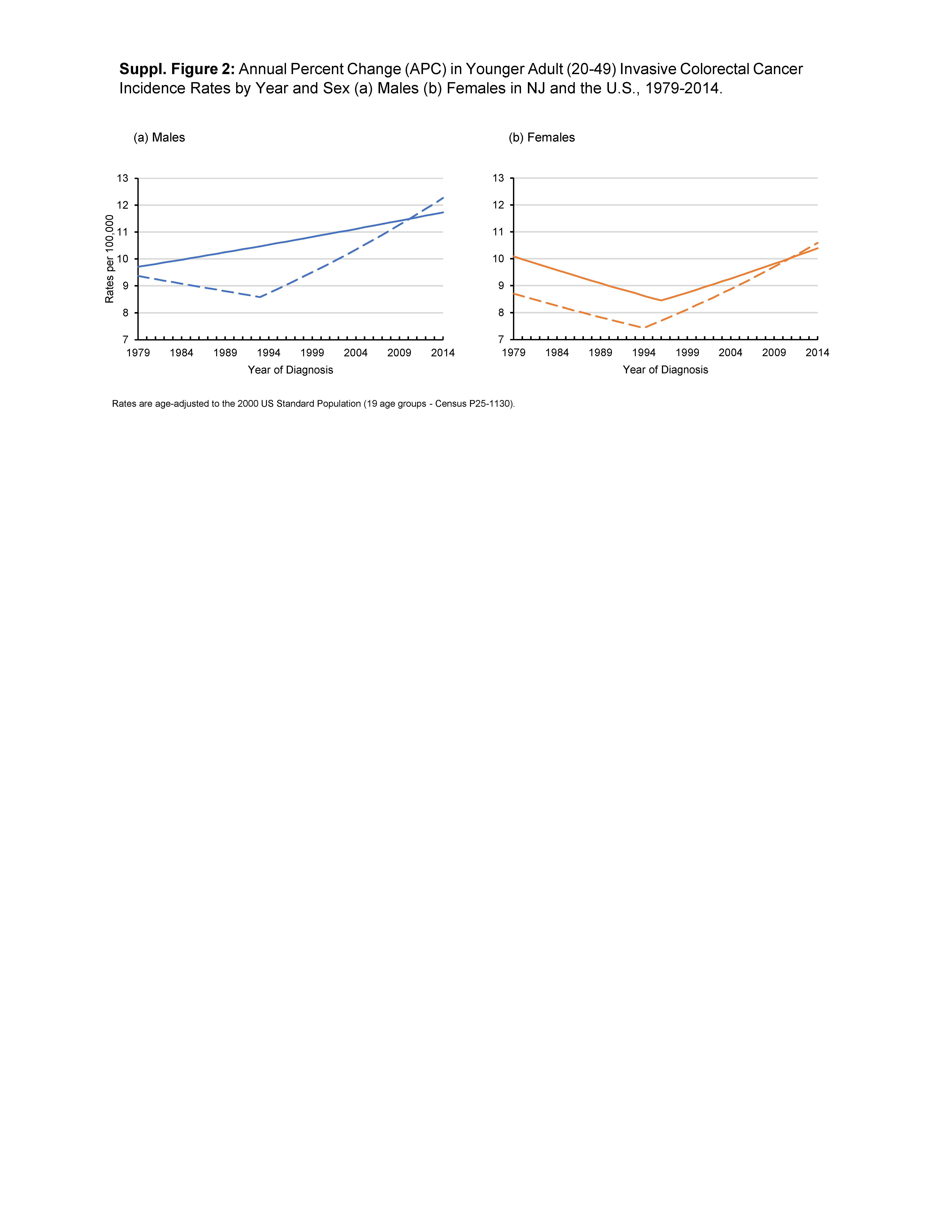

Supplement: Supplementary file 2 [file CAM4-7-4077-s002.tiff]
