# Supplementary material for: Trends in colorectal cancer incidence among younger adults—Disparities by age, sex, race, ethnicity, and subsite
Source: Cancer Med. 2018 Jun 22;7(8):4077–86. doi: 10.1002/cam4.1621 (PMC6089150; doi:10.1002/cam4.1621)
Supplement: Supplementary file 3 [file CAM4-7-4077-s003.docx]

| **Suppl Table 1:** Modifiable Risk Factors and the Strength of Association with CRC by Subsite | | | |
| --- | --- | --- | --- |
| **Risk Factor** | **Subsite** | | |
|  | **Distal** | **Proximal** | **Rectal** |
| Alcohol Use^45,51^ | *++* | *+* | *++* |
| Smoking^51^ | *+* | *++* | *+* |
| Red Meat^45,51^ | *++* | *+* | *++* |
| Processed Meat^45,52^ | *++* | *+* | *++* |
| High Body Mass Index^36^ | *++* | *+* | *+* |
| Physical Activity^36^ | *Inverse* | *Inverse* | *Not established* |
| *Helicobacter pylori*^53^ | *Possible +* | *Not established* | *Not established* |

Key: + Findings support the established positive association between the risk factor and CRC subsite, although not as strong as the ++ risk factor/subsite relationship. ++ Findings suggest a stronger positive association between the risk factor and CRC subsite. *Inverse*: Findings indicate a protective effect between the risk factor and CRC subsite. *Possible+*: Relationship remains controversial. *Not established*: Findings do not establish an effect for the subsite.

51. Wei EK, Colditz GA, Giovannucci EL, et al. A comprehensive model of colorectal cancer by risk factor status and subsite using data from the nurses’ health study. *Am J Epidemiol*. 2017;185:224‐237.

52. Bernstein AM, Song M, Zhang X, et al. Processed and unprocessed red meat and risk of colorectal cancer: analysis by tumor location and modification by time. *PLoS ONE*. 2015;10:e0135959.

53. Zhang Y, Hoffmeister M, Weck MN, Chang-Claude J, Brenner H. Helicobacter pylori and colorectal cancer risk: evidence from a large population-based case-control study in Germany. *Am J Epidemiol*. 2012;175:441‐450.
