# Supplementary material for: Trends in colorectal cancer incidence among younger adults—Disparities by age, sex, race, ethnicity, and subsite
Source: Cancer Med. 2018 Jun 22;7(8):4077–86. doi: 10.1002/cam4.1621 (PMC6089150; doi:10.1002/cam4.1621)
Supplement: Supplementary file 4 [file CAM4-7-4077-s004.docx]

**Suppl. Table 2**: Annual Percent Change (APC) in Adult Invasive Colorectal Cancer Incidence Rates by Year and Age Group in NJ and the U.S., 1979-2014.

| **Characteristic** | | **New Jersey, 1979-2014** | | | | | | | **United States (SEER 9), 1979-2014** | | | | | | |
| --- | --- | --- | --- | --- | --- | --- | --- | --- | --- | --- | --- | --- | --- | --- | --- |
|  |  | ***n*** | **Years** | **Rate (95% CI)** | **APC** | **Years** | **Rate (95% CI)** | **APC** | ***n*** | **Years** | **Rate (95% CI)** | **APC** | **Years** | **Rate (95% CI)** | **APC** |
| **Total (20-49):** | | **12,080** | **1979-1996** | **9.6 (9.3-9.9)** | **-0.5** | **1996-2014** | **10.2 (10.0-10.4)** | **+1.1*** | **34,333** | **1979-1994** | **8.4 (8.3-8.6)** | **-0.7*** | **1994-2014** | **9.6 (9.5-9.8)** | **+1.8*** |
| **Age** | 20-29 | 521 | 1979-2014 | 1.3 (1.2-1.4) | +2.1* | -- | -- | -- | 1,592 | 1979-2014 | 1.1 (1.1-1.2) | +2.6* | -- | -- | -- |
|  | 30-39 | 2,502 | 1979-2014 | 5.7 (5.5-5.9) | +1.1* | -- | -- | -- | 7,397 | 1979-1988 | 4.4 (4.2-4.6) | -1.0 | 1988-2014 | 5.5 (5.4-5.7) | +2.0* |
|  | 40-49 | 9,057 | 1979-2014 | 21.5 (21.1-22.0) | +0.1 | -- | -- | -- | 25,344 | 1979-1994 | 18.7 (18.3-19.2) | -1.1* | 1994-2014 | 20.5 (20.2-20.8) | +1.6* |
| **Total (50+):** | | **169,829** | **1979-1985** | **239.1 (236.5-241.7)** | **+2.3*** | **1985-1990** | **250.7 (247.9-253.5)** | **-0.8** | **414,381** | **1979-1985** | **213.3 (211.8-214.9)** | **+1.0*** | **1985-1995** | **199.8 (198.7-200.9)** | **-1.9*** |
|  |  |  | **1990-1994** | **230.8 (228.0-233.7)** | **-2.8*** | **1994-2001** | **214.7 (212.6-216.8)** | **-0.6** |  | **1995-1998** | **183.8 (182.2-185.5)** | **+1.3** | **1998-2008** | **164.5 (163.6-165.4)** | **-2.7*** |
|  |  |  | **2001-2010** | **169.8 (168.2-171.4)** | **-4.7*** | **2010-2014** | **130.3 (128.4-132.2)** | **-2.3*** |  | **2008-2011** | **131.8 (130.5-133.0)** | **-5.7*** | **2011-2014** | **116.6 (115.5-117.7)** | **-2.2*** |
|  | 50-59 | 24,368 | 1979-1985 | 81.9 (79.6-84.2) | +1.6* | 1985-1994 | 78.6 (76.6-80.6) | -2.3* | 66,127 | 1979-1985 | 72.9 (71.5-74.2) | +1.4 | 1985-1988 | 72.1 (70.3-73.9) | -4.3 |
|  |  |  | 1994-2001 | 71.2 (69.3-73.2) | +0.6 | 2001-2010 | 63.4 (62.0-64.9) | -3.0* |  | 1988-2014 | 62.0 (61.5-62.6) | -0.5* | -- | -- | -- |
|  |  |  | 2010-2014 | 57.6 (55.8-59.5) | +2.0 | -- | -- | -- |  | -- | -- | -- | -- | -- | -- |
|  | 60-69 | 44,368 | 1979-1985 | 211.2 (207.1-215.4) | +2.3* | 1985-2001 | 203.8 (201.2-206.5) | -1.4* | 107,939 | 1979-1985 | 184.2 (181.8-186.6) | +1.2* | 1985-1995 | 176.4 (174.5-178.2) | -1.6* |
|  |  |  | 2001-2009 | 142.7 (139.7-145.7) | -5.8* | 2009-2014 | 107.2 (104.4-110.1) | -1.8 |  | 1995-2000 | 163.2 (160.8-165.6) | +0.2 | 2000-2008 | 139.0 (137.3-140.7) | -3.9* |
|  |  |  | -- | -- | -- | -- | -- | -- |  | 2008-2012 | 106.2 (104.5-108.0) | -5.6* | 2012-2014 | 95.4 (93.3-97.4) | +0.5 |
|  | 70-79 | 56,915 | 1979-1985 | 406.3 (398.8-413.8) | +2.2* | 1985-2001 | 381.8 (377.6-386.1) | -1.5* | 132,380 | 1979-1985 | 351.5 (347.2-355.9) | +1.2* | 1985-1995 | 328.3 (325.3-331.3) | -2.0* |
|  |  |  | 2001-2014 | 254.0 (250.2-257.8) | -4.8* | -- | -- | -- |  | 1995-1999 | 298.8 (294.8-302.9) | +0.6 | 1999-2008 | 263.2 (260.5-265.9) | -3.3* |
|  |  |  | -- | -- | -- | -- | -- | -- |  | 2008-2014 | 188.5 (185.8-191.2) | -5.6* | -- | -- | -- |
|  | 80+ | 44,178 | 1979-1989 | 544.0 (544.0-564.3) | +2.2* | 1989-1993 | 557.2 (543.5-571.2) | -4.7* | 107,935 | 1979-1988 | 500.6 (494.6-506.8) | -0.1 | 1988-1995 | 454.4 (448.7-460.2) | -2.5* |
|  |  |  | 1993-2000 | 505.6 (496.2-515.1) | -0.0 | 2000-2014 | 378.3 (373.0-383.7) | -4.1* |  | 1995-1998 | 431.0 (423.8-438.4) | +1.8 | 1998-2008 | 372.1 (368.3-375.9) | -3.3* |
|  |  |  | -- | -- | -- | -- | -- | -- |  | 2008-2014 | 273.5 (269.8-277.4) | -4.6* | -- | -- | -- |

Rates are age-adjusted to the 2000 US Standard Population (19 age groups - Census P25-1130); Confidence intervals (Tiwari mod) are 95% for rates.
