# Supplementary material for: Trends in colorectal cancer incidence among younger adults—Disparities by age, sex, race, ethnicity, and subsite
Source: Cancer Med. 2018 Jun 22;7(8):4077–86. doi: 10.1002/cam4.1621 (PMC6089150; doi:10.1002/cam4.1621)
Supplement: Supplementary file 5 [file CAM4-7-4077-s005.docx]

**Suppl. Table 3:** Annual Percent Change (APC) in Younger Adult (20-49) Invasive Colorectal Cancer Incidence Rates by Year and Sex.

| **Characteristic** | | **New Jersey, 1979-2014** | | | | | | |
| --- | --- | --- | --- | --- | --- | --- | --- | --- |
|  |  | ***n*** | **Years** | **^Rate (95% CI)** | **APC** | **Years** | **Rate (95% CI)** | **APC** |
| **Sex** | Males | 6,353 | 1979-2014 | 10.7 (10.4-11.0) | +0.5* | -- | -- | -- |
|  | Females | 5,727 | 1979-1996 | 9.1 (8.8-9.5) | -1.0* | 1996-2014 | 9.3 (9.0-9.6) | +1.2* |
| **Characteristic** | | **United States (SEER 9), 1979-2014** | | | | | | |
|  |  | ***n*** | **Years** | **Rate (95% CI)** | **APC** | **Years** | **Rate (95% CI)** | **APC** |
| **Sex** | Males | 18,231 | 1979-1993 | 8.9 (8.7-9.2) | -0.6 | 1993-2014 | 10.3 (10.1-10.5) | +1.7* |
|  | Females | 16,102 | 1979-1994 | 7.9 (7.7-8.2) | -1.0* | 1994-2014 | 8.9 (8.7-9.1) | +1.8* |

Rates are age-adjusted to the 2000 US Standard Population (19 age groups - Census P25-1130); Confidence intervals (Tiwari mod) are 95% for rates. *APC is statistically significant (p< 0.05).
