# Supplementary material for: Trends in colorectal cancer incidence among younger adults—Disparities by age, sex, race, ethnicity, and subsite
Source: Cancer Med. 2018 Jun 22;7(8):4077–86. doi: 10.1002/cam4.1621 (PMC6089150; doi:10.1002/cam4.1621)
Supplement: Supplementary file 6 [file CAM4-7-4077-s006.docx]

| **Characteristic** | | **New Jersey, 1992-2014** | | | | | | | |
| --- | --- | --- | --- | --- | --- | --- | --- | --- | --- |
|  |  | **Males** | | | | **Females** | | | |
|  |  | ***n*** | **Years** | **Rate (95% CI)** | **APC** | ***n*** | **Years** | **Rate (95% CI)** | **APC** |
| **Race^a^** | White | 3,595 | 1992-2014 | 10.9 (10.6-11.3) | +1.2* | 2,947 | 1992-2014 | 8.8 (8.5-9.1) | +1.1* |
|  | Black | 667 | 1992-2014 | 12.0 (11.1-12.9) | -0.4 | 777 | 1992-2014 | 11.9 (11.1-12.8) | +0.3 |
|  | API | 254 | 1992-2014 | 7.8 (6.9-8.8) | +2.0* | 223 | 1992-2014 | 6.5 (5.7-7.5) | +0.2 |
|  | Hispanic (of any race) | 571 | 1992-2014 | 9.3 (8.5-10.1) | +0.5 | 554 | 1992-2014 | 9.0 (8.3-9.8) | -1.5* |
| **Characteristic** | | **United States (SEER 13), 1992-2014** | | | | | | | |
|  |  | **Males** | | | | **Females** | | | |
|  |  | ***n*** | **Years** | **Rate (95% CI)** | **APC** | ***n*** | **Years** | **Rate (95% CI)** | **APC** |
| **Race^a^** | White | 13,711 | 1992-2014 | 9.3 (9.2-9.5) | +1.8* | 11,558 | 1992-2014 | 8.1 (7.9-8.2) | +2.0* |
|  | Black | 2,470 | 1992-2014 | 12.5 (12.0-13.0) | +0.3 | 2,551 | 1992-2014 | 11.2 (10.8-11.7) | 0.0 |
|  | API | 2,470 | 1992-2014 | 10.9 (10.5-11.4) | +0.9* | 2,270 | 1992-2014 | 9.0 (8.7-9.4) | -0.1 |
|  | Hispanic (of any race) | 2,729 | 1992-2014 | 7.6 (7.3-7.9) | +2.0* | 2,466 | 1992-2014 | 7.1 (6.8-7.4) | +1.1* |

**Suppl. Table 4**: Annual Percent Change (APC) in Younger Adult (20-49 years) Colorectal Cancer Incidence Rates by Race/Ethnicity and Year (a) NJ Males, 1992-2014.

Rates are per 100,000 and age-adjusted to the 2000 US Standard Population (19 age groups - Census P25-1130). An asterisk denotes that the APC is statistically significant (p< 0.05).
