# Supplementary material for: Trends in colorectal cancer incidence among younger adults—Disparities by age, sex, race, ethnicity, and subsite
Source: Cancer Med. 2018 Jun 22;7(8):4077–86. doi: 10.1002/cam4.1621 (PMC6089150; doi:10.1002/cam4.1621)
Supplement: Supplementary file 7 [file CAM4-7-4077-s007.docx]

**Suppl. Table 5:** Annual Percent Change (APC) in Younger Adult (20-49 Years) Invasive Colorectal Cancer Incidence Rates by Subsite, Age Group, Sex, Race and Year in NJ, 1979-2014.

| Characteristic | Proximal | | | | Distal | | | | Rectal | | | | | | |
| --- | --- | --- | --- | --- | --- | --- | --- | --- | --- | --- | --- | --- | --- | --- | --- |
|  | *n* | Years | Rates^ (95% CI) | APC | *n* | Years | Rates^ (95% CI) | APC | *n* | Years | Rates^ (95% CI) | APC | Years | Rates^ (95% CI) | APC |
| Age 20-49 | 3,168 | 1979-2014 | 2.6 (2.5-2.7) | +0.5* | 3,614 | 1979-2014 | 3.0 (2.9-3.1) | 0.0 | 4,563 | 1979-2014 | 3.8 (3.6-3.9) | +1.3* | -- | -- | -- |
| 20-39 | 829 | 1979-2014 | 1.0 (0.9-1.1) | +1.1* | 836 | 1979-2014 | 1.0 (0.9-1.1) | +1.3* | 1,149 | 1979-2014 | 1.4 (1.3-1.5) | +2.1* | -- | -- | -- |
| 40-49 | 2,339 | 1979-2014 | 5.6 (5.3-5.8) | +0.3 | 2,778 | 1979-2014 | 6.6 (6.4-6.9) | -0.4 | 3,414 | 1979-2014 | 8.1 (7.9-8.4) | +1.0* | -- | -- | -- |
| Sex Female | 1,474 | 1979-2014 | 2.4 (2.3-2.5) | +0.6* | 1,866 | 1979-2014 | 3.0 (2.9-3.1) | -0.2 | 2,054 | 1979-2014 | 3.3 (3.2-3.5) | +0.8* | -- | -- | -- |
| Male | 1,694 | 1979-2014 | 2.9 (2.7-3.0) | +0.4 | 1,748 | 1979-2014 | 3.0 (2.8-3.1) | +0.2 | 2,509 | 1979-1986 | 3.5 (3.1-3.9) | +7.1* | 1986-1991 | 3.6 (3.2-4.0) | -7.4 |
|  | -- | -- | -- | -- | -- | -- | -- | -- | -- | 1991-2000 | 3.8 (3.5-4.1) | +5.3* | 2000-2014 | 5.0 (4.7-5.3) | +0.8 |
| Race White | 2,366 | 1979-2014 | 2.4 (2.3-2.5) | +0.5* | 2,837 | 1979-2014 | 2.9 (2.8-3.0) | +0.3 | 3,573 | 1979-1991 | 3.2 (3.0-3.4) | -0.3 | 1991-2014 | 3.9 (3.8-4.1) | +2.0* |
| Black | 660 | 1979-2014 | 4.0 (3.7-4.3) | +0.7 | 557 | 1979-2014 | 3.4 (3.1-3.7) | -1.2* | 650 | 1979-2014 | 3.9 (3.6-4.2) | -- | -- | -- | +0.6* |

Rates are age-adjusted to the 2000 US Standard Population (19 age groups - Census P25-1130). An asterisk denotes that the APC is statistically significant (p< 0.05).
